# Traditional Chinese medicine monomers: a new strategy against diabetic kidney disease by regulating cholesterol efflux

**DOI:** 10.3389/fnut.2025.1708809

**Published:** 2025-11-25

**Authors:** Chenguang Wu, Jianing Sun, Junwei Shi, Zhe Li, Yan An, Han Zhu, Lifan Wang, Peng Liu

**Affiliations:** 1 Renal Division, Department of Medicine, Heilongjiang Academy of Chinese Medicine Sciences, Harbin, China; 2 Xiyuan Hospital, China Academy of Chinese Medical Sciences, Beijing, China; 3 Jiangxi University of Chinese Medicine, Nanchang, Jiangxi, China

**Keywords:** diabetic kidney disease, cholesterol efflux, traditional Chinese medicine, ABCA1, PPARs

## Abstract

Diabetic kidney disease (DKD), a prevalent microvascular complication of diabetes, is driven by a complex pathogenesis. A key pathological hallmark of early DKD is intrarenal lipid deposition, a process pivotally driven by impaired cholesterol efflux. This efflux is critically mediated by ATP-binding cassette (ABC) transporters, primarily ABCA1 and ABCG1. Dysfunction of these transporters precipitates cholesterol accumulation in renal cells, subsequently inducing oxidative stress, inflammation, and fibrosis. Recently, a diverse range of herbal monomers has emerged as a promising class of therapeutic agents for DKD. Compounds—including Anthocyanins, Morroniside, Resveratrol, Tanshinone, Puerarin, Baicalin, Curcumin, Protocatechuic acid, and Kaempferol—have been shown to activate the PPARγ and LXRα signaling pathways. This activation upregulates the expression of ABCA1 and ABCG1, thereby enhancing cholesterol efflux, mitigating renal lipid deposition, and ultimately slowing DKD progression. However, this body of research is largely limited to preclinical studies *in vitro* and in animal models. Consequently, the complex, multi-target mechanisms of these compounds *in vivo* remain poorly understood. Future investigations should therefore leverage multi-omics technologies to comprehensively delineate these mechanisms. Furthermore, large-scale clinical trials are imperative to validate the therapeutic efficacy and safety of these agents, potentially establishing novel strategies for the prevention and treatment of DKD.

## 1 Introduction

Diabetic kidney disease (DKD), a major microvascular complication of diabetes, is a leading cause of end-stage renal disease (ESRD). Affecting approximately one-third of individuals with diabetes worldwide, the incidence of DKD is escalating in parallel with the global diabetes pandemic (1–3). The disease is clinically characterized by an initial presentation of proteinuria that progresses to nephrotic syndrome and, ultimately, renal failure (4). While its progression is driven by multiple factors, including persistent hyperglycemia and hypertension, dyslipidemia has emerged as a particularly critical and modifiable driver, making its management a key therapeutic strategy (5). However, current therapeutic regimens, which primarily target glycemic and blood pressure control, have proven insufficient to halt disease progression in many patients (6, 7).

Growing evidence implicates intrarenal lipid accumulation as a central pathogenic mechanism in DKD (8–10). Specifically, this accumulation is driven by impaired cholesterol efflux—the process of transporting intracellular cholesterol to extracellular acceptors like apolipoprotein A-I (apoA-I) via ATP-binding cassette (ABC) transporters (11, 12). The transporter ABCA1 is a master regulator of this process; its dysfunction leads to cholesterol overload in podocytes and tubular epithelial cells. This lipid toxicity triggers a cascade of deleterious events, including oxidative and endoplasmic reticulum stress (ERS), inflammation, and ultimately, renal fibrosis (13, 14). This pathogenic cascade is further substantiated by evidence linking ABCA1 dysfunction to cardiolipin-driven mitochondrial damage and by clinical observations where reduced cholesterol efflux in DKD patients accelerates disease progression (15, 16). Preclinical validation comes from animal models, where podocyte-specific Abca1 knockout exacerbates glomerulosclerosis, whereas treatment with ABCA1 agonists like cyclodextrin ameliorates DKD pathology (14). Collectively, these findings establish the impairment of ABCA1-mediated cholesterol efflux as a critical, early event in DKD pathogenesis.

**Figure 1:**
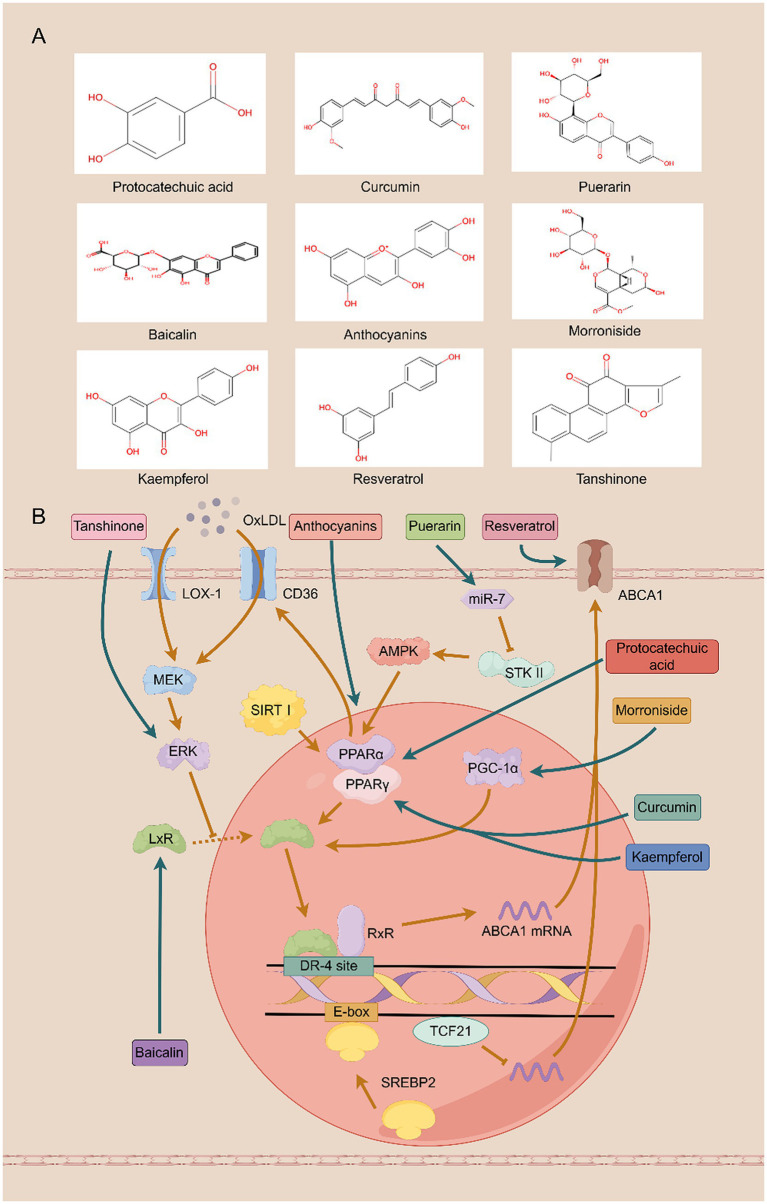
Traditional Chinese medicine monomers protect against diabetic kidney disease by improving cholesterol efflux. **(A)** The structure of the compound. **(B)** The mechanism involved in cholesterol efflux.

Given this complex pathophysiology, therapeutic strategies capable of addressing multiple targets are highly sought after. Traditional Chinese medicine (TCM) offers a promising source of such agents, valued for its multi-component, multi-target nature and favorable safety profiles (17–19). Indeed, isolated monomers from TCM have shown efficacy in DKD by modulating diverse pathological processes, including inflammation, hyperglycemia, and insulin resistance (20–23). Critically, a key emerging mechanism is their ability to regulate cholesterol efflux by modulating ABC transporter expression. Despite growing interest, a systematic synthesis of this specific mechanism is currently lacking. This review, therefore, consolidates and analyzes the existing evidence on how TCM-derived monomers modulate ABC transporters to treat DKD, providing a mechanistic framework summarized in Table 1 and Figure 1.

**Table 1:** Traditional Chinese medicine monomers that regulate the cholesterol efflux process.

Classification	Monomer	Experimental model	Signal pathway	References
Directly acting on the ABC process to treat DKD	Cyanidin	HK-2 cells	PPARα/LXRα/ABCA1	(28)
	Morroniside	mRTECs; mice podocyte	PGC-1α/LXR/ABCA1/ABCG1	(11, 33)
Potential therapeutic effects	Resveratrol	THP-1 macrophage	PPARγ/ABCA1	(37)
	Tanshinone	THP-1 macrophage, primary human macrophages	ERK/Nrf2/HO-1/ ABCA1/ ABCG1	(43)
	Puerarin	THP-1 macrophage	STK11 / AMPK-PPARγ/LXR-α/ABCA1	(48)
	Baicalin	THP-1 macrophage	PPARγ/LXRα/ABCA1/ABCG1	(52)
	Curcumin	podocyte	CXCL16/PPARγ/LXRα/ABCA1	(55–57)
	Protocatechuic acid	mice peritoneal macrophage, THP-1 macrophage	miRNA-10b/ABCA1/ABCG1	(60, 61)
	Kaempferol	Primary hepatocytes	PPARγ/LXRα/ABCA1	(65)

## 2 Traditional Chinese medicine monomers regulate cholesterol efflux

### 2.1 Treatment of DKD by direct action

Anthocyanins, a class of pleiotropic flavonoids widely found in plants, manifest potent renal protective effects by modulating inflammation, oxidative stress, and metabolic homeostasis (24). Mechanistically, they suppress the nuclear factor kappa B (NF-κB) signaling pathway, which in turn attenuates the expression of pro-inflammatory mediators, including tumor necrosis factor-*α* (TNF-α), interleukin-6 (IL-6), and monocyte chemotactic protein-1 (MCP-1) in renal tissues. This anti-inflammatory action has been validated *in vitro*, where anthocyanins dose-dependently inhibit palmitic acid-induced cytokine expression and NF-κB activity in human renal tubular epithelial cells (25). Furthermore, anthocyanins counteract oxidative stress and fibrosis by upregulating core components of the glutathione (GSH) antioxidant system (GCLC, GCLM) while concurrently inhibiting key fibrotic signaling molecules such as transforming growth factor-β1 (TGF-β1), *α*-smooth muscle actin (α-SMA), and Collagen IV (26). In the context of glucose homeostasis, they modulate intestinal absorption by inhibiting sodium-glucose cotransporter 1 (SGLT1) and glucose transporter 2 (GLUT2) and enhance systemic glucose disposal by promoting AMP-activated protein kinase (AMPK)-mediated glucose transporter 4 translocation, thereby improving insulin sensitivity (27). Building on these metabolic benefits, recent work has identified a critical role for anthocyanins in regulating lipid metabolism. These compounds activate the peroxisome proliferator-activated receptor alpha (PPARα) / liver X receptor alpha (LXRα) signaling axis, leading to the upregulation of ABCA1. This, in turn, promotes cholesterol efflux, reduces intrarenal lipid deposition, and slows the progression of renal fibrosis. Crucially, the functional necessity of this pathway was confirmed in experiments where pharmacological or genetic inhibition of either PPARα or LXRα completely abrogated the protective effects of anthocyanins (28).

Morroniside, the principal bioactive iridoid glycoside derived from the medicinal herb *Cornus officinalis*, exhibits a spectrum of organ-protective functions, including potent nephroprotection (29). Its renoprotective effects are multifaceted. Within podocytes, it restores cellular homeostasis by modulating autophagy via mechanistic target of rapamycin (mTOR) inhibition and by suppressing NOX4-mediated reactive oxygen species (ROS) accumulation (30). Concurrently, morroniside counteracts glomerulosclerosis by blocking the interaction between advanced glycation end products (AGEs) and their receptor (RAGE). This action disrupts downstream pro-fibrotic signaling cascades (p38/MAPK/NF-κB/TGF-*β*), thereby curtailing mesangial cell proliferation and extracellular matrix deposition (31). Beyond these established mechanisms, morroniside fundamentally reshapes the renal lipid landscape in DKD by downregulating the master lipid regulators sterol regulatory element-binding protein 1/2 (SREBP-1/2) (32). It orchestrates a dual strategy to combat lipid accumulation. First, it promotes cholesterol efflux by activating the peroxisome proliferator-activated receptor gamma coactivator 1-alpha (PGC-1α)/LXRα signaling axis, leading to the transcriptional upregulation of the transporters ABCA1 and ABCG1 (11). Second, it simultaneously curtails cholesterol uptake by engaging the PGC-1α/PPARγ pathway to suppress the expression of the fatty acid translocase CD36 (33). This synergistic regulation—enhancing efflux while inhibiting influx—corrects intracellular lipid imbalance, preserves podocyte integrity, and robustly delays the progression of DKD.

### 2.2 Traditional Chinese medicine monomers with potential therapeutic value

Resveratrol, an extensively studied natural polyphenol found in medicinal herbs such as *Polygonum cuspidatum* and in dietary sources like grapes, exerts pleiotropic renoprotective effects (34). A primary mechanism involves its engagement of the pivotal metabolic regulators AMPK and sirtuin 1 (SIRT1), which in turn activate PGC-1α. This signaling cascade orchestrates a multi-pronged defense against lipotoxicity by upregulating adiponectin receptors 1 and 2 (AdipoR1/2) to reduce intracellular fatty acids and triglycerides, while simultaneously suppressing pro-apoptotic pathways (PI3K/Akt/FOXO3a) and bolstering antioxidant defenses (SOD1/2, BCL-2) (35, 36). Critically, beyond managing lipotoxicity, Resveratrol directly targets the machinery of cholesterol efflux. Resveratrol dose-dependently upregulates the expression of the key transporters ABCA1 and ABCG1, as well as the cholesterol-metabolizing enzyme 27-hydroxylase. This effect is mediated through the PPARγ signaling axis, a conclusion supported by experiments where the upregulation was abrogated by PPARγ antagonists (37). The convergence of these pathways—mitigating lipid-induced damage while actively promoting cholesterol removal—translates into significant attenuation of key DKD pathologies, including proteinuria, glomerular matrix expansion, and inflammation.

*Salvia miltiorrhiza* is a traditional Chinese medicinal herb with a long history of use. Tanshinones represent the most pharmacologically active class of compounds in *Salvia miltiorrhiza* (38), encompassing multiple related compounds such as Tanshinone I, Tanshinone IIA, and Tanshinone IIB (39). Tan IIA is known to exert broad renoprotective effects by concurrently mitigating inflammation, oxidative stress, endoplasmic reticulum (ER) stress, and pyroptosis. These actions collectively reduce renal fibrosis and preserve kidney function. Tanshinone IIA (Tan IIA) reduces inflammatory responses in the kidneys by inhibiting the expression of TGF-β1, MCP-1, and P-selectin, thereby decreasing the production of inflammatory mediators. It also alleviates oxidative stress induced by diabetes through lowering malondialdehyde (MDA) levels and increasing superoxide dismutase (SOD) activity, thus protecting the kidneys from oxidative damage (40). Animal studies demonstrate that Tan IIA also alleviates endoplasmic reticulum (ER) stress by inhibiting activation of the protein kinase RNA-like ER kinase (PERK) signaling pathway. This prevents phosphorylation of eukaryotic translation initiation factor 2 alpha, reduces levels of the transcription factor activating transcription factor 4 (ATF4), thereby downregulating TGF-β1 expression and decreasing extracellular matrix (ECM) accumulation, ultimately mitigating renal fibrosis in diabetic nephropathy (41). Furthermore, Tan IIA inhibits NOD-like receptor family pyridine domain-containing protein 3 (NLRP3), reduces cleavage of caspase-1 and interleukin-1β (IL-1β), indicating Tan IIA mitigates pyroptosis in human renal glomerular endothelial cells (HRGECs) by suppressing NLRP3 inflammasome activation (42). Recent studies reveal that Tan IIA inhibits scavenger receptor A (SR-A)-mediated uptake of oxidized low-density lipoprotein (oxLDL) and enhances ABCA1/G1-mediated cholesterol efflux by activating the ERK/Nrf2/HO-1 pathway and suppressing mRNA rapid decay, thereby mitigating foam cell formation damage to normal cells (43). This dual action—blocking lipid influx while promoting efflux—prevents the formation of lipid-laden foam cells within the renal microenvironment. This regulation of cholesterol trafficking is now understood as a critical mechanism through which Tan IIA preserves renal cell integrity and slows the progression of DKD.

Puerarin, the primary active component extracted from Pueraria root, belongs to the isoflavone class. As a natural antioxidant, Puerarin possesses significant health benefits, exhibiting a range of biological activities including antioxidant, anti-inflammatory, antitumor effects, immune enhancement, and protection of cardiovascular and neural cells (44, 45). *In vitro* experiments demonstrated that Puerarin significantly reduced the expression of pro-inflammatory cytokines IL-1β, IL-6, IL-18, and TNFα, with the most pronounced inhibition observed for IL-1β. *In vivo* studies revealed that Puerarin suppresses vascular inflammation, mitigates vascular calcification, and delays chronic kidney disease progression by inhibiting NLRP3 inflammasome activation, thereby reducing Caspase1 activation and IL-1β production (46). Furthermore, Puerarin enhances NF-κB inhibition by activating SIRT1 to reduce p65 acetylation, thereby suppressing NF-κB-mediated expression of other inflammatory factors and protecting glomerular function. Combining Puerarin with arctigenin potentiates their renal protective effects (47). Building on this established anti-inflammatory profile, recent studies have elucidated a sophisticated molecular mechanism by which Puerarin directly regulates lipid homeostasis. Puerarin downregulates microRNA-7 (miR-7), a microRNA that post-transcriptionally silences serine/threonine kinase 11 (STK11) by targeting its 3′ untranslated region (3′ UTR). This de-repression of STK11 unleashes the downstream AMPK/PPARγ/LXRα signaling cascade, culminating in the transcriptional upregulation of ABCA1. The resulting enhancement of cholesterol efflux from macrophages effectively reduces intracellular lipid accumulation and lipotoxicity (48). This microRNA-mediated regulation of cholesterol trafficking represents a key mechanism by which Puerarin may mitigate the lipotoxic component of DKD.

Baicalin (BAI), a principal flavonoid isolated from Scutellaria baicalensis, is recognized for its potent renoprotective properties, which stem from its ability to concurrently disarm pro-inflammatory signaling and neutralize oxidative stress (49). BAI inhibits activation of the MAPK signaling pathway by reducing the phosphorylation levels of extracellular signal-regulated kinase 1/2 (ERK1/2), c-Jun N-terminal kinase (JNK), and p38 mitogen-activated protein kinase (p38) phosphorylation levels. This downregulates mRNA levels of inflammatory cytokines (IL-1β, IL-6, MCP-1, and TNFα), thereby alleviating renal fibrosis (50). Concurrently, it bolsters cellular antioxidant defenses by upregulating key enzymes such as SOD and GSH-px, thereby mitigating oxidative damage (51). Crucially, the therapeutic efficacy of BAI in DKD is now understood to be critically dependent on its direct regulation of cholesterol homeostasis. *In vitro* studies have demonstrated that BAI drives the expression of the master lipid regulators PPARγ and LXRα at both the mRNA and protein levels. This, in turn, transcriptionally upregulates the cholesterol transporters ABCA1 and ABCG1 to facilitate robust cholesterol efflux and ameliorate cellular lipotoxicity. The indispensability of this axis is confirmed by gene-silencing experiments, where siRNA-mediated knockdown of either PPARγ or LXRα completely abrogated the BAI-induced upregulation of ABCA1 and ABCG1 (52). Therefore, Baicalin’s capacity to simultaneously resolve inflammation and correct dysregulated lipid metabolism positions it as a compelling therapeutic candidate for DKD.

*Turmeric*, a spice native to India, has a long history of use in China. Curcumin, an active polyphenolic compound extracted from turmeric, exhibits anti-inflammatory, anticancer, anti-aging, and blood glucose-regulating effects (53, 54). It effectively neutralizes multiple reactive oxygen species and suppresses the master inflammatory transcription factor NF-κB, providing a broad cellular defense against metabolic stressors (55). In the specific context of DKD, a key mechanism underlying its renoprotective action involves its ability to thwart the pathogenic actions of the chemokine CXCL16 in podocytes. Under diabetic conditions, CXCL16 drives lipotoxicity by concurrently suppressing the lipid sensor PPARγ and upregulating the pro-inflammatory enzyme cyclooxygenase-2 (COX2). Curcumin intervenes directly by inhibiting CXCL16 expression, an action that prevents podocyte lipid accumulation and apoptosis (56). The centrality of this target is underscored by gene-silencing studies, where CXCL16 knockdown not only phenocopied Curcumin’s protective effects but also completely abrogated its regulatory influence on PPARγ and COX2. This CXCL16-mediated rescue of PPARγ subsequently unleashes the canonical LXRα-ABCA1 signaling axis, culminating in the enhanced expression of the ABCA1 transporter and its partner, apoA-I, to drive cholesterol efflux (55, 57). Therefore, Curcumin’s capacity to dismantle this specific, lipotoxic CXCL16-PPARγ signaling cascade illuminates a critical pathway through which it restores podocyte lipid homeostasis and slows DKD progression.

Protocatechuic acid (PCA) is a naturally occurring phenolic acid widely present in our daily diet and herbal medicines. It is also one of the primary metabolites of complex polyphenols such as Anthocyanins and proanthocyanidins (58). Protocatechuic acid exerts functions including antioxidant, anti-inflammatory, and protective effects on the cardiovascular system, liver, and kidneys (59). Recent mechanistic studies have pinpointed a sophisticated regulatory role for PCA in cholesterol homeostasis. In pathological contexts, microRNA-10b (miRNA-10b) acts as a post-transcriptional repressor of the cholesterol transporters ABCA1 and ABCG1, driving lipid accumulation and cellular lipotoxicity. PCA directly counteracts this process by suppressing the expression of miRNA-10b. This de-repression restores the functional expression of ABCA1 and ABCG1, thereby re-establishing efficient cholesterol efflux from macrophages (60). This targeted regulation of lipid metabolism complements PCA’s systemic benefits observed in diabetic animal models. There, PCA administration not only improves key metabolic parameters—reducing blood glucose and BUN while enhancing insulin levels and creatinine clearance—but also directly modulates renal gene expression. Specifically, it restores the expression of the crucial lipid sensors PPARα and PPARγ and suppresses the expression of the pro-inflammatory receptor for advanced glycation end-products (RAGE) (61). Therefore, PCA’s therapeutic potential in DKD stems from a dual-front attack: its ability to precisely target the miRNA-10b/ABCA1 axis to resolve cellular lipotoxicity and its capacity to concurrently improve systemic glycemic control and suppress renal inflammation.

Kaempferol (KPF), a prominent flavonoid abundant in various medicinal herbs, is renowned for a spectrum of therapeutic properties, including potent anti-diabetic and renoprotective activities (62, 63). At the level of renal histopathology, KPF directly counters the structural degradation characteristic of DKD. *In vivo* studies demonstrate that KPF reverses key pathological hallmarks, including mesangial matrix expansion, podocyte foot process effacement, and glomerular basement membrane thickening. This structural preservation is driven by its ability to restore cellular homeostasis. KPF recalibrates the central energy sensor AMP-activated protein kinase (AMPK), which in turn restrains the anabolic mTOR pathway to suppress aberrant cell growth and unleashes protective autophagy, a critical cellular quality control mechanism (64). A significant portion of KPF’s efficacy is mediated by its active metabolite, kaempferol-3-O-gallate, which orchestrates a dual correction of the dysregulated lipid and glucose metabolism central to DKD. It systematically improves the lipid profile and enhances insulin sensitivity in diabetic models. Mechanistically, this metabolite stimulates cholesterol efflux by activating the canonical PPARγ/LXRα/ABCA1 signaling cascade. Concurrently, it enhances insulin signaling by engaging a distinct PPARγ-PI3K/AKT pathway to improve cellular glucose uptake (65). Therefore, the therapeutic potential of kaempferol in DKD arises from a sophisticated, dual-component mechanism: the parent flavonoid preserves renal architecture and cellular integrity via the AMPK-autophagy axis, while its key metabolite systemically corrects the underlying metabolic pathologies of lipid and glucose dysregulation.

## 3 Summary and outlook

The chronic lipotoxic and glucotoxic milieu of DKD progressively suppresses the expression of pivotal cholesterol transporters, thereby trapping lipids within renal cells and driving a relentless cycle of cellular injury and disease progression. The evidence presented herein delineates a compelling therapeutic strategy: the targeted reactivation of this cholesterol efflux machinery by a diverse pharmacopeia of herbal monomers. By engaging key regulatory nodes such as the PPARγ/LXRα axis, these natural compounds restore the function of ABCA1/G1, offering a powerful mechanism to mitigate lipotoxicity and preserve renal function.

However, translating this preclinical promise into clinical reality requires surmounting critical knowledge gaps. Current research, while mechanistically insightful, has been largely constrained to a “single-target, single-pathway” paradigm, focusing predominantly on the ABCA1 axis. This approach fails to capture the holistic, multi-target synergy that likely underpins the efficacy of these pleiotropic compounds. Current studies have also revealed several limitations of natural products, particularly their poor oral bioavailability and rapid metabolic clearance. Future research may address these issues through the use of advanced delivery systems such as nanocarriers (e.g., solid lipid nanoparticles and SEDDS), phospholipid complexes (phytosomes), or co-administration with metabolic inhibitors like piperine to markedly enhance bioavailability (66–68). Furthermore, the field has certain limitations in terms of preclinical research models. In *in vitro* experiments, immortalized cell lines (e.g., HK-2 cells) and primary cells derived from non-diabetic individuals fail to replicate the chronic stress state associated with human DKD. In animal experiments, streptozotocin-induced diabetic models and db/db mouse models exhibit notable differences from humans in both pathological characteristics and physiological mechanisms. More critically, the severity and progression rate of human DKD are significantly influenced by age, comorbidities, and genetic background, and existing models fail to recapitulate the heterogeneity of human DKD. In the future, priority should be given to the adoption of humanized models, combined with multi-omics technologies, to enhance the reliability of research findings.

The path forward must therefore be twofold. First, a paradigm shift toward systems-level inquiry is essential. The application of multi-omics technologies (e.g., transcriptomics, proteomics, metabolomics) is imperative to comprehensively map the network-wide effects of these compounds *in vivo*, deciphering their true multi-target mechanisms beyond the established ABC transporter axis. Secondly, it is necessary to conduct large-scale screening of traditional Chinese medicine monomers that have been proven to have cholesterol efflux regulatory activity, in order to identify candidate compounds with significant therapeutic effects, good pharmacokinetic characteristics, and low toxicity. And validate its efficacy and safety in the humanized DKD model to better simulate human pathological states. Upon establishing robust preclinical evidence of these network-level effects, the ultimate validation must come from rigorously designed, large-scale, multicenter, randomized controlled trials. These trials are indispensable for definitively assessing the clinical efficacy and safety of the most promising candidates. Such a strategy will not only illuminate novel biology but also pave the way for a new class of evidence-based, mechanism-driven therapeutics for the prevention and treatment of DKD.
